# Benchmarking DNA extraction protocols across use cases for culture-independent Nanopore metagenomics

**DOI:** 10.1099/mgen.0.001738

**Published:** 2026-07-15

**Authors:** Tara Signorelli, Matthew Walker, James Robertson, Kaye Quizon, Yongxiang Zhang, Aleisha R. Reimer, Shannon H.C. Eagle

**Affiliations:** 1 National Microbiology Laboratory, Public Health Agency of Canada, Guelph, Ontario, Canada; 2 National Microbiology Laboratory, Public Health Agency of Canada, Winnipeg, Manitoba, Canada

**Keywords:** DNA extraction, long-read sequencing, metagenomics, Oxford Nanopore sequencing, public health

## Abstract

Oxford Nanopore Technologies (ONT) sequencing offers several advantages for metagenomics, including long reads, rapid turnaround, low upfront cost, scalability and portability. However, for ONT metagenomics, DNA yield, quality and integrity are important considerations when selecting an extraction method. Many metagenomic extraction methods use harsh lysis conditions to extract a wide range of species and provide an accurate community composition, but these conditions can compromise DNA fragment length. Therefore, extraction methods for ONT metagenomics must balance DNA shearing and recovery with representative community lysis. We systematically evaluated DNA extraction methods for ONT metagenomic sequencing using a use case-oriented framework. Among nearly 50 extraction methods screened, 7 were selected for detailed comparison based on suitability for metagenomics, variation in methodology, availability, cost and processing time: Norgen BioTek Corp’s Stool DNA Isolation (NG), Zymo Research’s ZymoBIOMICS Quick-DNA HMW MagBead (ZMG), Qiagen’s DNeasy Blood and Tissue (QBT), Macherey-Nagel’s NucleoMag DNA Microbiome (MN), Zymo Research’s ZymoBIOMICS DNA Mini Prep (ZMI), Qiagen’s DNeasy PowerSoil/QIAamp PowerFecal Pro (PS) and Qiagen’s QIAamp Fast DNA Stool Mini (QIA). Methods were tested using Zymo Research’s ZymoBIOMICS Microbial Community Standard (MCS), a matrix-free mock community with known composition. DNA extracts were sequenced on an ONT PromethION using the Rapid Barcoding Kit, except QIA due to insufficient DNA yield. Metrics for the method, DNA extracts, sequencing and genomes were evaluated, revealing trade-offs between methods. The two magnetic bead methods, MN and ZMG, produced the highest mean read length N50 values (13.9 and 16.5 kb, respectively) but showed apparent community compositions skewed towards Gram-negative bacteria. In contrast, ZMI and PS maintained a community composition close to expected, with reduced mean read length N50 values (4.5 vs. 7.5 kb). Performance across various metrics is presented in the context of the following use cases: maximizing genome coverage and assembly completeness, preserving composition accuracy, targeting specific species and limiting required resources (equipment, time or budget). The metrics and use case considerations presented offer practical guidance for informed selection of DNA extraction methods for ONT metagenomics. For accurate community composition, ZMI or PS are recommended, while PS and ZMG perform best at maximizing genome coverage and assembly completeness. NG and QBT may be the most economical options, though performance trade-offs were observed. Finally, PS may be the preferred method for time-sensitive diagnostic or field applications.

**Impact Statement**

Oxford Nanopore Technologies (ONT) sequencing provides many benefits for metagenomics. Long reads lead to longer contigs in metagenome-assembled genomes. In clinical metagenomics, the ability to process in real time allows targeted patient care to start sooner. The portability of ONT devices enables sample processing on-site, eliminating transportation costs and logistical challenges. Despite these advantages, few studies have explored the impact of DNA extraction methods on ONT metagenomic sequencing outcomes. Previous work with isolates has suggested that DNA extraction methods can impact sequence read length and output with ONT, but evaluations for metagenomic samples have focused on single use cases. This study adds to this body of knowledge by evaluating seven metagenomic extraction methods using a wide variety of qualitative metrics, providing a framework for future method testing. By linking method outcomes to different metagenomic use cases, recommendations provided help laboratories select appropriate methods and reduce barriers to implementing ONT metagenomics.

## Data Summary

Basecalled sequence data (FASTQ files) have been uploaded to NCBI BioProject PRJNA1270948.

Supplementary Material – Excel: Table S1 – List of extraction methods considered. Table S2 – Minimap2 alignment of reads – gaps output. Table S3 – Minimap2 alignment of MAGs – gaps output. Table S4 – Extraction, sequencing and genome metrics for all replicates for each extraction method. Table S5 – Sequencing and genome metrics for the extraction methods sequenced on ONT by replicate and by species. Table S6 – Percentage of nonzero coverage for plasmids for the extraction methods sequenced on ONT by replicate.

PNG: Fig. S1 – Heatmap of gaps from reads aligned to reference genome. Fig. S2 – Heatmap of gaps from assemblies aligned to reference genome.

The authors confirm that all supporting data, code and protocols have been provided within the article or through supplementary data files.

## Introduction

Metagenomics is widely used for diverse applications, for example, human microbiome studies [1], species discovery [2], environmental monitoring [3] and clinical diagnostics [4–6]. Ideally, the sequencing technology used for metagenomics applications generates long reads, as these enable the assembly of more resolved and complete metagenome-assembled genomes (MAGs) [7] and enhance precision for species-level classification [8]. In addition to long reads, Oxford Nanopore Technologies (ONT) devices provide other advantages, including a range of sizes, throughput and portability, all while offering real-time data analysis and the ability to detect methylated nucleotides [9, 10]. Real-time ONT sequencing runs can be stopped once sufficient data is obtained, enabling flow cell reuse and cost reductions [11]. However, unlike Illumina library preparation workflows, many ONT protocols do not employ an amplification step and therefore require high input DNA amounts (200–1,000 ng). As a result, extraction methods for ONT should be evaluated for their ability to deliver sufficient yield without introducing inhibitory contaminants, while simultaneously preserving long DNA fragments.

Previous studies have demonstrated that DNA extraction methods can have a substantial impact on the sequence read lengths obtained with ONT whole-genome sequencing (WGS) [12, 13]. Chemical and heat lysis produced sufficiently long DNA fragments for the generation of complete, closed genomes for *Salmonella enterica*, a Gram-negative bacterium [12], while *Streptococcus mitis*, a Gram-positive bacterium, required enzymatic lysis, as mechanical methods caused DNA shearing and led to reduced read lengths [13]. Consistent with these organism-specific findings, Kruasuwan *et al*. [14] determined that no single commercial extraction method performed well across eight individual pathogenic bacteria they tested with ONT-only WGS.

DNA extraction methods have also been shown to impact inferred species composition when evaluating mixed community samples. Several Illumina-based studies have evaluated these effects on stool or sewage samples, determining that the extraction method used impacted the estimated bacterial community compositions [15] and has a greater impact than library preparation and sample storage conditions [16]. Additionally, the DNA extraction method affects the recovery of both bacterial and fungal community members [17]. ONT metagenomic studies have similarly reported extraction-dependent variation in sequencing outcomes, although most have relied on evaluations focused on specific organisms or performance metrics, limiting guidance for readers with different analytical objectives. For example, Gand *et al*. [18] prioritized recovery of the longest reads from mixtures of *Bacillus subtilis* and *Escherichia coli*, while Zhang *et al*. [19] evaluated pathogen detection in clinical urine samples, and Purushothaman *et al*. [20] assessed antimicrobial gene detection from clinical samples and mock communities. Together, these studies highlight the need for a broader, use-case-oriented evaluation of DNA extraction methods for ONT metagenomic sequencing.

In this study, six DNA extraction methods were comprehensively evaluated for ONT metagenomics. Four DNA replicates from Zymo Research’s commercially available ZymoBIOMICS Microbial Community Standard (MCS) were extracted using Norgen BioTek Corp’s Stool DNA Isolation kit (NG), Zymo Research’s ZymoBIOMICS Quick-DNA HMW MagBead kit (ZMG), Qiagen’s DNeasy Blood and Tissue kit (QBT), Macherey-Nagel’s NucleoMag DNA Microbiome kit (MN), Zymo Research’s ZymoBIOMICS DNA Mini Prep Kit (ZMI) and Qiagen’s DNeasy PowerSoil/QIAamp PowerFecal Pro kit (PS). The ONT Rapid Barcoding Kit was used to prepare these DNA extracts for sequencing on PromethION flow cells. DNA extracts from a seventh kit, Qiagen’s QIAamp Fast DNA Stool Mini (QIA), were not sequenced due to their low yield. The MCS was chosen because it includes several well-characterized enteric pathogens in a standardized community containing a variety of Gram-positive and Gram-negative bacteria and two yeast species, which allows for detailed comparisons against a known ground truth. Methods were evaluated based on metrics associated with the method (methodology, time, bead beating and cost), DNA (concentration and quality), sequencing (read quality, sequencing yield, composition, read length N50 and read length N50 variability by species) and genomes (depth and breadth of coverage, genome fraction, mismatches, number of contigs and plasmid coverage). These metrics were used to evaluate the suitability of the DNA extraction methods for a variety of common analytical use cases: maximizing genome coverage and assembly completeness, preserving composition accuracy, targeting specific species and limiting required resources (equipment, time or budget).

## Methods

### Extraction kit selection and costing

Selections were made from a list of nearly 50 extraction methods, most of which are commercially available kits (Table S1, available in the online Supplementary Material); however, there are a few with modified protocols. Modifications were based on in-house development and/or derived from publications [e.g. direct boiling, Bhatt high molecular weight (HMW) from stool [21], THSTI [22] and Three Peaks [23]]. The Three Peaks Protocol from the University of Birmingham [23] uses Zymo Research’s ZymoBIOMICS Quick-DNA HMW MagBead kit with modifications to reduce DNA shearing. Qiagen’s DNeasy Blood and Tissue kit, Omega’s E.Z.N.A. Bacterial DNA kit and Epicentre’s MasterPure Complete DNA and RNA Purification kit do not have metagenomic samples listed as a possible matrix but were considered as they are used for isolates in our laboratory and were part of a comparison study on DNA extraction kits for ONT WGS [12]. The QIAamp DNA Stool Mini kit from Qiagen was previously Protocol Q [16], the recommended microbiome protocol from International Human Microbiome Standards (IHMS) [24]; however, Qiagen has replaced this kit with the QIAamp Fast DNA Stool Mini kit, and the current IHMS Protocol Q is QIAsymphony DSP Virus/Pathogen Midi kit using the QIAsymphony SP instrument [25]. Finally, a few modification options were available for Epicentre’s MasterPure Complete DNA and RNA Purification kit (in-house vs. manufacturer vs. extra reagents), Qiagen’s QIAamp Fast DNA Stool Mini kit (various bead-beating options) and Zymo Research’s ZymoBIOMICS Quick-DNA HMW MagBead kit (manufacturer protocol vs. Three Peaks Protocol [23]). Selection criteria included the following: manufacturer-recommended use for metagenomics, manufacturer matrix recommendations (i.e. stool listed as a sample type), no host depletion step, including a variety of methodologies (at least one kit of each of column and magnetic bead), cost, ease of procurement, time to perform, scalability, feasibility for automation and/or high-throughput (HTP) and history (used previously in our group or commonly in microbiome work). Using these criteria, seven methods were selected, based on the following kits: NG (Norgen BioTek Corp, Cat# 27600), ZMG (Zymo Research, Cat# D6060), QBT (Qiagen, Cat# 69504), MN (Macherey-Nagel, Cat# 744330.1), ZMI (Zymo Research, Cat# D4300), PS (Qiagen, Cat# 47014 or 51804) and QIA (Qiagen, Cat# 51604) (Table 1). Methods either use columns (PS, ZMI, QBT, NG and QIA) or magnetic beads (MN, ZMG) to separate DNA from contaminants and cell debris (Table 1). The selected methods include bead-beating times of none (QBT, ZMG), 5 min (NG), 10 min (PS, MN and QIA) and 40 min (ZMI) (Table 1).

**Table 1.** Summary of key metrics for the seven extraction methods tested. Extraction, sequencing and genome metrics are the mean (±sd) for four replicates of the Zymo Research ZymoBIOMICS MCS. Times are for simultaneous extraction of four replicates, where total time is the time taken to go from sample material to purified DNA, and hands-on time only includes when the technician is actively engaged in the process. Costs were calculated based on list prices in Canada on 2 February 2026. N.T., not tested; n/a, not applicable

Metric	Kit name	Stool DNA Isolation (*n*=4)	ZymoBIOMICS Quick-DNA HMW MagBead (*n*=4)	DNeasy Blood and Tissue (*n*=4)	NucleoMag DNA Microbiome (*n*=4)	ZymoBIOMICS DNA Mini Prep (*n*=4)	DNeasy PowerSoil/QIAamp PowerFecal Pro (*n*=4)	QIAamp Fast DNA Stool Mini (*n*=4)
Details	Abbreviation	NG	ZMG	QBT	MN	ZMI	PS	QIA
	Manufacturer	Norgen BioTek Corp.	Zymo Research	Qiagen	Macherey-Nagel	Zymo Research	Qiagen	Qiagen
	Catalogue number	27600	D6060	69504	744330.1	D4300	47014/51804	51604
	Reactions/kit	50	96	50	96	50	50/50	50
	Lysis methodologies employed	Mechanical, Chemical+heat	Enzymatic+heat, Chemical	Enzymatic+heat, Chemical	Mechanical, Chemical+heat	Mechanical, Chemical	Mechanical, Chemical	Enzymatic+heat, Mechanical, Chemical+heat
	Bead-beating time (min)	5	0	0	10	40	10	10
	DNA isolation methodology	Spin column	Magnetic bead	Spin column	Magnetic bead	Spin column	Spin column	Spin column
Method	Total time (min)	70	180	120	140	95	40	50
	Hands-on time (min)	70	120	60	130	55	30	40
	Cost per extraction (CAD)	6.82	14.13	6.11	10.57	10.48	16.38	9.87
Extraction	Concentration (ng µl^−1^)	7.5 ± 0.4	23.3 ± 2.2	19.9 ± 1.5	12.5 ± 0.6	25.8 ± 1.0	38.5 ± 4.6	2.2 ± 0.2
	260/280 absorbance ratio	2.04 ± 0.06	1.82 ± 0.14	2.16 ± 0.06	1.86 ± 0.06	1.97 ± 0.03	1.88 ± 0.02	N.T.
	260/230 absorbance ratio	1.33 ± 0.07	1.63 ± 0.37	1.39 ± 0.24	1.04 ± 0.29	1.12 ± 0.26	0.57 ± 0.22	N.T.
Sequencing	Mean read quality	13.5 ± 0	13.0 ± 0.04	13.5 ± 0.07	13.5 ± 0	13.8 ± 0.04	13.3 ± 0.07	n/a
	Sequencing yield (Gbp)	1.3 ± 0.2	6.5 ± 2.3	7.9 ± 1.5	7.6 ± 2.4	4.1 ± 0.7	5.8 ± 1.3	n/a
	Composition (MIQ score)	2 ± 0.4	33 ± 2.1	0 ± 0	3 ± 1.8	81 ± 0.8	67 ± 0.8	n/a
	Read length N50 (kbp)	9.8 ± 0.3	16.5 ± 0.8	13.2 ± 0.2	13.9 ± 0.4	4.5 ± 0.2	7.5 ± 0.2	n/a
	Deviation in read length N50 between species (kbp)	8.9 ± 0.4	4.1 ± 0.8	9.7 ± 0.6	6.8 ± 0.7	0.7 ± 0.08	1.4 ± 0.1	n/a
Genome (eight bacterial species only)	No. of species >40× coverage	3.0 ± 0.7	7.5 ± 0.5	4.0 ± 0	5.3 ± 1.6	8.0 ± 0	8.0 ± 0	n/a
	No. of species >1× at 99% bp	4.0 ± 0	7.8 ± 0.4	7.0 ± 0	8.0 ± 0	8.0 ± 0	8.0 ± 0	n/a
	Genome fraction (%)	72.5 ± 2.3	97.8 ± 0.4	90.0 ± 0.5	97.8 ± 0.06	97.4 ± 0.02	97.7 ± 0.03	n/a
	No. of mismatches per 100 kbp	50.1 ± 11.8	2.3 ± 2.1	7.9 ± 2.3	2.2 ± 1.5	11.6 ± 2.7	3.0 ± 0.7	n/a
	No. of species with LGA50 ≤1	3.8 ± 0.4	7.5 ± 0.5	6.5 ± 0.9	7.8 ± 0.4	5.8 ± 0.4	7.5 ± 0.5	n/a
	No. of contigs aligned to reference	93 ± 15	15 ± 3	27 ± 4	15 ± 4	56 ± 5	21 ± 3	n/a

The costs of each method were calculated based on list prices in Canada in Canadian dollars (CAD) on 2 February 2026 and include prices for kits, enzymes and additional bead tubes but do not account for required additional chemicals (ethanol, SDS), generic plasticware (tips, tubes), personal protective equipment or labour (Table 1).

### Extraction of MCS DNA

All seven methods were tested by extracting DNA from four technical replicates of the ZymoBIOMICS MCS (Zymo Research, Cat# D6300). The MCS is provided in DNA/RNA Shield (Zymo Research, Cat# R1100) and is composed of a known composition of ten species, eight bacteria and two yeast, as follows: *Listeria monocytogenes* (12%), *Pseudomonas aeruginosa* (12%), *B. subtilis* (12%), *E. coli* (12%), *S. enterica* (12%), *Lactobacillus fermentum* (12%), *Enterococcus faecalis* (12%), *Staphylococcus aureus* (12%), *Saccharomyces cerevisiae* (2%) and *Cryptococcus neoformans* (2%) [26]. According to the reference genome file provided by Zymo Research (https://zymo-files.s3.amazonaws.com/BioPool/ZymoBIOMICS.STD.refseq.v3.zip), there are a total of seven plasmids present in the MCS: one each in *B. subtilis*, *E. coli* and *P. aeruginosa* genomes and two each in *S. enterica* and *S. aureus* genomes. The reference genome files used in this study for the MCS were provided by Zymo Research.

DNA extractions were performed on 75 µl of thawed MCS. Bead-beating steps were completed on a horizontal vortex adapter (Scientific Industries, Inc., Cat# SI-H524) at maximum speed. In most cases, modifications to the manufacturer’s protocol were made only if they were suggested in the FAQ or troubleshooting sections of the protocol or handbook; all modifications are listed below.

#### Norgen Stool DNA Isolation (NG)

As recommended in the protocol, Lysis Buffer L was reduced to 800 µl to accommodate the sample volume. After bead beating with the proprietary bead tube provided, the samples were incubated at 65 °C for 10 min at 300 r.p.m., as per the Troubleshooting Guide and with clarification from Norgen BioTek Corp Technical Support. The samples were incubated with Binding Buffer I at 4 °C in a fridge instead of on ice. DNA was eluted in 50 µl of Elution Buffer B, and filters were incubated for 3 min at 37 °C immediately prior to the final spin to enhance recovery.

#### ZymoBIOMICS Quick-DNA HMW MagBead (ZMG)

This kit uses magnetic beads and avoids mechanical lysis to maximize recovery of HMW DNA. Instruction Manual Appendix A, ‘Cells and Solids’ steps were followed, as recommended for microbial/environmental sample types including stool. In step 6 of ‘Microbial Lysis’, 100 µl of 1× PBS and 10 µl of MetaPolyzyme (Cat# MAC4L-5MG, Sigma) were added instead of Tris-EDTA buffer and lysozyme. MetaPolyzyme is recommended in the Three Peaks Protocol [23] to enhance lysis capabilities for Gram-positive bacteria and yeast [27], and in accordance with Sigma’s protocol, the sample was incubated for 1 h at 35 °C. Finally, for step 16, the air-dry option was performed.

#### DNeasy Blood and Tissue kit (QBT)

DNA extraction followed the ‘Purification of Total DNA from Animal Tissues (Spin-Column) Protocol’, using a lysis time of 1 h and the optional RNase step (5 µl of 100 mg ml^−1^ RNase incubated at 37 °C for 5 min). For steps 2 and 3, Buffer AL and ethanol were combined prior to addition to the sample (as recommended in the Qiagen kit handbook for the QBT 96 protocol), and samples were mixed by pipetting.

#### NucleoMag DNA Microbiome (MN)

Macherey-Nagel MN Bead Tubes Type A (Cat# 740786.5), which contain 0.6–0.8 mm ceramic beads [28], were the recommended bead tubes and had to be purchased separately. The following recommendations from Macherey-Nagel for improved lysis efficiency and DNA recovery were incorporated into the protocol: the optional 5-min incubation at 70 °C, a maximum recommended mechanical disruption time during sample preparation and the optional mix by pipetting in each step.

#### ZymoBIOMICS DNA Mini Prep (ZMI)

Tubes of ZR Bashing Beads of 0.1 and 0.5 mm diameters are provided in the ZMI kit [29], and bead beating was done for 40 min as recommended by the protocol when using a horizontal vortex adaptor. DNA was eluted in 50 µl of DNase/RNase free water, and filters were incubated for 3 min at 37 °C prior to spinning.

#### DNeasy PowerSoil/QIAamp PowerFecal Pro (PS)

Provided bead tube composition is proprietary. The Qiagen protocol recommends a final elution volume of 50–100 µl of C6 Buffer; DNA was eluted in 50 µl.

#### QIAamp Fast DNA Stool Mini (QIA)

The ‘Isolation of DNA from Stool for Pathogen Detection Protocol’ was followed, with modifications informed by the work of Knudsen *et al*. [15], the manufacturer’s recommendations and prior experience. These modifications included the addition of a 10-min bead-beating step using MP Biomedicals Lysing Matrix E (Cat# 116914050), composed of 1.4 mm ceramic spheres, 0.1 mm silica spheres and one 4 mm glass bead [30], after the first heating step. Reagent volumes were doubled in steps 5 to 7, and in step 10, the addition of a second application of lysate and centrifugation to accommodate the increased volume. DNA was eluted in 50 µl of DNase/RNase free water, and filters were incubated for 3 min at 37 °C prior to the final spin.

While performing extractions, the time required to complete each method from sample to purified DNA was recorded (total time). Hands-on time was defined as the time the operator was actively engaged in the procedure; incubation or bead-beating steps of 10 min or longer were excluded, as the operator would be free to perform a different task.

Recovered DNA from all methods was quantified using the Qubit dsDNA Broad Range Assay Kit (Invitrogen, Cat# Q32853). Current versions of the ONT Rapid Barcoding Kit recommend at least 200 ng DNA in a volume of 10 µl, i.e. 20 ng µl^−1^ [31]; however, previous versions of the protocol allowed as little as 50 ng in 10 µl, i.e. 5 ng µl^−1^ [32]. Further testing was only done if the method could produce a minimum concentration of 5 ng µl^−1^, and as a result, QIA replicates were excluded from subsequent analyses.

DNA purity was assessed by measuring absorbance ratios (260/280 and 260/230) using the NanoDrop 8000 spectrophotometer. ONT recommends a 260/280 absorbance ratio of 1.8, which represents pure DNA without RNA contamination, and 260/230 ratio between 2.0 and 2.2, representing DNA free from organic materials [33].

### ONT sequencing and data analysis

Sequencing libraries were prepared using the ONT Rapid Barcoding Kit (Cat# SQK-RBK110.96) following the manufacturer’s protocols. Sequencing was done on PromethION R9.4.1 flow cells (ONT, Cat# FLO-PRO002) for 72 h with live basecalling and demultiplexing using Guppy v6.4.6 integrated into MinKNOW v22.12.5. Sequencing runs generated between 100 and 145 gigabases (Gbp) of data.

Reads were analysed using NanoComp v1.19.3 [34], Measurement Integrity Quotient (MIQ) score v1.2.0 [35] and K-mer Analysis Toolkit (KAT) v2.4.2 [36]. Reference genomes for the MCS from Zymo Research were used when required for these analyses. Modifications were made to the MIQ score script to run in long-read mode with MCS as the sample type. The script provides percent composition by species and a MIQ score. The MIQ score is out of 100, representing deviations from the expected composition, with a lower score indicating greater deviations [35, 37]. NanoComp was run by replicate and used to directly evaluate the sequencing yield (Gbp) and mean read length N50 values. KAT sect and KAT filter were used to assign reads by species within a replicate. KAT sect was used to determine the nonzero coverage, which is the percent of the genome that has sequence read coverage greater than 1, also referred to as breadth of coverage. Finally, NanoComp v1.20.0 was run on the reads assigned to each species within a replicate; this data was used to calculate the deviation in read length N50 between species (subtracting the smallest species mean read length N50 from the largest, for each replicate) and crude genome coverage depth (dividing total bases generated by reference genome size). Forty-fold coverage is recommended by PulseNet International as a minimum for WGS of *E. coli* and *Shigella* spp. isolates sequenced with Illumina platforms [38] and was used in this study as a target.

Metagenomes were assembled using metaFlye v2.9.5 [39] with the meta and nano-raw options and were evaluated using MetaQUAST v5.3.0 [40] with reference genome files for the MCS. metaFlye [39] has been shown to be effective and consistent when handling ONT data [7, 12]. MetaQUAST metrics were evaluated at both the replicate and species levels. Replicate-level metrics included genome fraction (how much of the reference genome is covered by at least one contig of the assembly) and number of mismatches per 100 kb (bases in the assembly that do not match the reference genome). Species and replicate-level metrics included LGA50 (minimum number of contigs required to cover 50% of the reference genome) and the number of bacterial contigs per assembly.

Additionally, both reads and metagenomic assemblies were aligned to the bacterial reference genomes for the MCS with minimap2 [41], with output alignments in SAM format; the ‘-ax -map-ont’ preset was applied to reads, and ‘-x -asm5’ to assemblies. Alignments were converted to BAM format using SAMtools view [42], excluding unaligned reads, secondary alignments and supplementary alignments from the output (SAM flag -F 2308). SAMtools sort was then applied before using Bedtools genomecov [43] to report the depth in a bedGraph format, including regions with 0 coverage (-bga). The Bedtools output was filtered using awk to identify all positions with 0 depth and tabulated (Tables S2 and S3).

Figures were generated in Microsoft Excel and PowerPoint or RStudio [44] using ggplot2 v4.0.1 [45].

## Results

### Extraction method selection

Nearly 50 extraction methods were considered (Table S1), most of which are commercially available kits. Methods were evaluated based on manufacturer-recommended use for metagenomics, manufacturer matrix recommendations, a lack of a host depletion step, methodology, cost, ease of procurement, time to perform, scalability, feasibility for automation and/or HTP and history. To identify methods best suited for use in a diagnostic laboratory focused on enteric pathogen identification from stool samples, seven kits were selected for laboratory testing: Norgen BioTek Corp’s Stool DNA Isolation (NG), Zymo Research’s ZymoBIOMICS Quick-DNA HMW MagBead (ZMG), Macherey-Nagel’s NucleoMag DNA Microbiome (MN), Zymo Research’s ZymoBIOMICS DNA Mini Prep (ZMI), Qiagen’s DNeasy PowerSoil/QIAamp PowerFecal Pro (PS), Qiagen’s QIAamp Fast DNA Stool Mini (QIA), and Qiagen’s DNeasy Blood and Tissue (QBT) (Table 1). NG was selected because it is inexpensive, from a Canadian company, and specific to stool. QBT, PS and QIA all had been used in our laboratory. QBT was previously used in performance testing on bacterial isolates [12], PS was being concurrently used for other metagenomics projects and QIA had been used for stool metagenomics with Illumina sequencing. QIA is also Qiagen’s replacement for the previous IHMS Protocol Q for microbiome studies. Although the IHMS now recommends QIAsymphony DSP Virus/Pathogen Midi Kit, it was not selected because selection criteria prioritized extraction kits that support manual and HTP and/or automated workflows. MN was chosen as it is a magnetic bead-based kit and is easily convertible to an HTP setting. ZMI claims to give accurate community composition, and according to its handbook, the MCS was used for kit validation. Finally, ZMG is another magnetic bead-based kit and is used as the basis for the Three Peaks Protocol [23] because of its ability to maintain long DNA fragments. Although the Three Peaks Protocol [23] was tried, early tests produced low DNA yields (<5 ng µl^−1^) and demonstrated that this method was impractical for diagnostic or reference laboratories, as it was laborious and took longer than a typical workday. Therefore, the manufacturer’s protocol for ZMG was chosen, with one modification borrowed from the Three Peaks method, using MetaPolyzyme instead of lysozyme.

### Method metrics

Method metrics evaluated were time, both total and hands-on and cost (Table 1). In terms of the total time taken from sample to isolated DNA for four samples extracted simultaneously, PS and QIA are the fastest at under 1 h; ZMI and NG are less than 2 h; and MN, QBT and ZMG are between 2 and 3 h (Table 1). Because of some longer incubations and/or bead-beating steps in certain methods, hands-on time is less than total time for MN, PS and QIA (↓10 min), ZMI (↓40 min) and QBT and ZMG (↓60 min) (Table 1).

Costs in CAD range from $6.11 to $16.38 per reaction (Table 1). The least expensive method is QBT; however, it is not intended for metagenomic samples. The least expensive metagenomic method is NG at $6.82/reaction. QIA is just under $10/reaction, while ZMI and MN are just over ($10.48 and $10.57, respectively, Table 1). ZMG and PS are the most expensive, both more than $14/reaction ($14.13 and $16.38, respectively, Table 1).

### DNA metrics

**Fig. 1.**
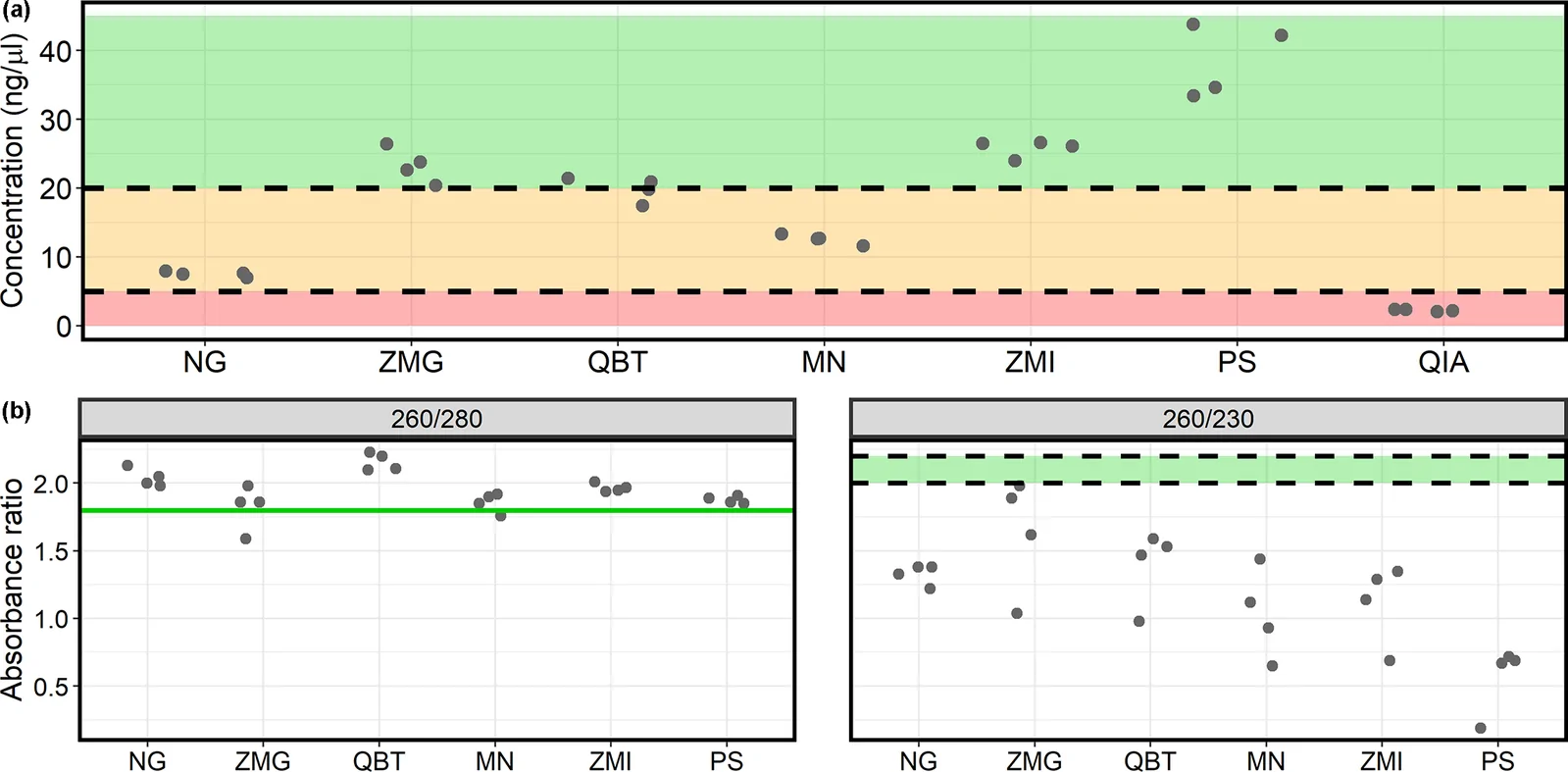
DNA quantity (**a**) and quality (**b**) for the seven extraction methods. The kits tested were Norgen BioTek Corp’s Stool DNA Isolation (NG), Zymo Research’s ZymoBIOMICS Quick-DNA HMW MagBead (ZMG), Qiagen’s DNeasy Blood and Tissue (QBT), Macherey-Nagel’s NucleoMag DNA Microbiome (MN), Zymo Research’s ZymoBIOMICS DNA Mini Prep Kit (ZMI), Qiagen’s DNeasy PowerSoil/QIAamp PowerFecal Pro (PS) and Qiagen’s QIAamp Fast DNA Stool Mini (QIA). Lines are shown at 5 and 20 ng µl^−1^ (**a**), 1.8 (b, 260/280) and 2.0 and 2.2 (b, 260/230). Green areas highlight ONT’s current recommendations for DNA quantity and quality.

Mean DNA concentrations measured by Qubit ranged from 2.2 to 38.5 ng µl^−1^, with most sd below 2.0 (Table 1). QIA had the lowest concentrations with no replicates exceeding 3 ng µl^−1^ (Fig. 1a, Table S4) and was excluded from further testing as it did not meet the lowest minimum concentration for ONT sequencing of 5 ng µl^−1^ [32]. NG was next lowest at 7.5 ng µl^−1^, followed by MN and QBT with means between 10 and 20 ng µl^−1^ (Table 1). The two Zymo methods (ZMI, ZMG) had means around 25 ng µl^−1^; however, ZMI was more consistent across replicates than ZMG (SD 1.0 vs. 2.2, Table 1, Fig. 1a, Table S4). PS had the highest concentration of all methods with a mean of almost 40 ng µl^−1^; however, the sd was also high (4.6, Table 1). The high sd for PS is caused by variability between replicates, with two in the mid-30s and two in the low 40s (Fig. 1a, Table S4).

A NanoDrop microvolume spectrophotometer was used to determine 260/280 and 260/230 absorbance ratios for DNA quality assessment. Mean 260/280 absorbance ratios for the methods ranged from 1.82 to 2.16 (Table 1). While most of the tested replicates had ratios above 1.8, there were two exceptions: a ZMG replicate at 1.59 and an MN replicate at 1.76 (Fig. 1b, Table S4). Mean 260/230 absorbance ratios ranged from 0.57 to 1.63, while replicates fell between 0.19 and 1.98 (Table 1, Fig. 1b, Table S4). As a result, none of the replicates met the ONT standard for clean DNA [33].

### Sequencing metrics

Sequencing metrics such as read quality, sequencing yield, composition, read length N50 and deviation in read length N50 between species were evaluated post-sequencing. Mean read qualities, or Q-scores, ranged from 13.0 for ZMG to 13.8 for ZMI, and were consistent between replicates for each method (sd ranged from 0 for NG and MN, up to 0.07 for PS and QBT) (Tables 1 and S4).

Based on the reference genome sizes provided for the MCS and expected relative abundance of each organism, it was assumed that the coverage of each member was equal and it was calculated that ~2 Gbp of data is required to reach 40× coverage for bacterial species [46]. Mean sequencing yield of more than 5.8 Gbp was achieved for four methods (ZMG, QBT, MN and PS), with some variation between replicates (sd=1.3 to 2.4, Tables 1 and S4). ZMI generated mean sequencing data of 4.1 Gbp and was consistent between replicates (sd=0.7, Tables 1 and S4). NG generated a mean of 1.3 Gbp of data, and none of the four replicates exceeded the 2 Gbp target (Tables 1 and S4).

**Fig. 2.**
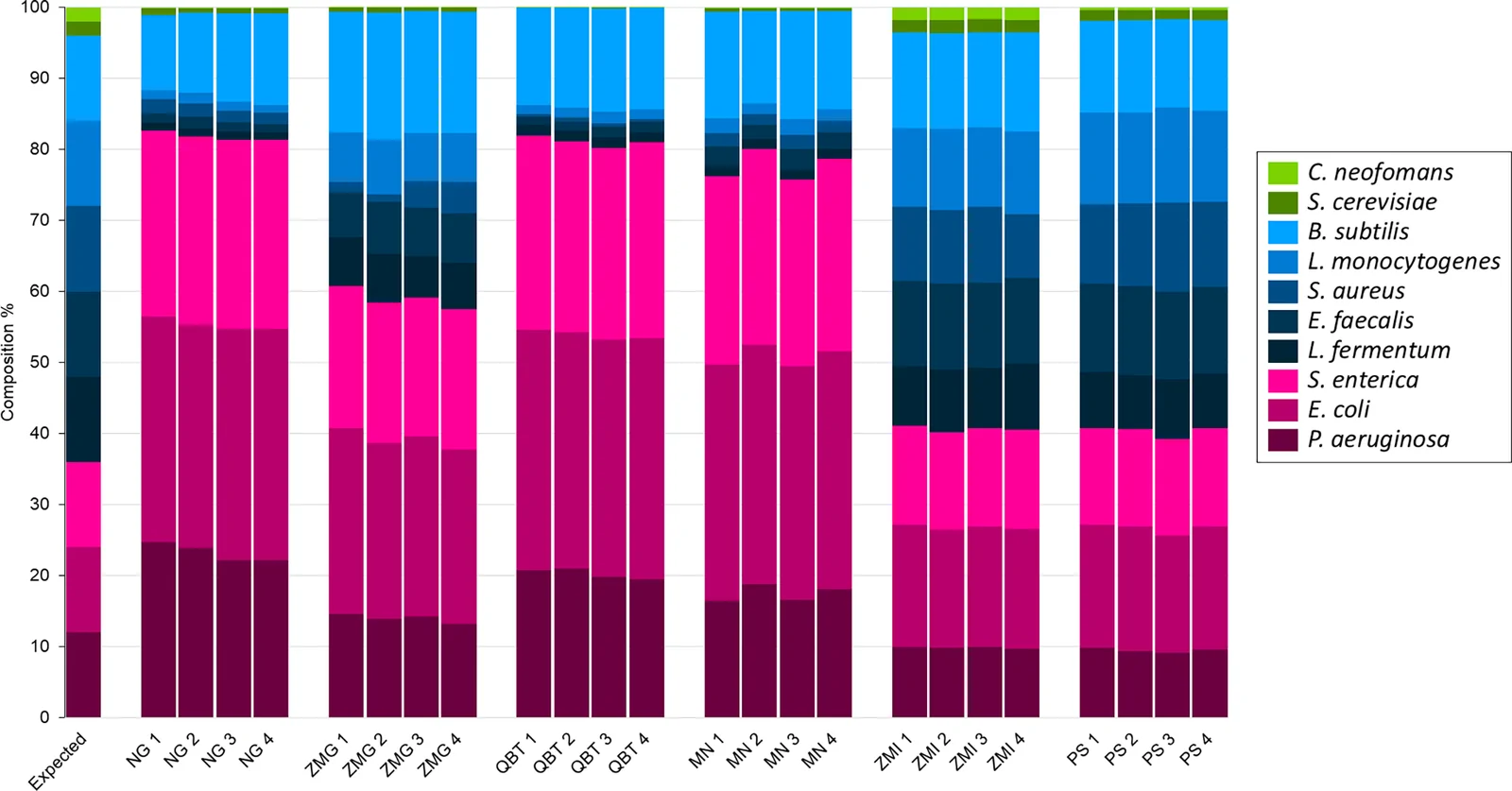
Species composition for the four replicates for each of the six extraction methods sequenced. The kits tested were Norgen BioTek Corp’s Stool DNA Isolation (NG), Zymo Research’s ZymoBIOMICS Quick-DNA HMW MagBead (ZMG), Qiagen’s DNeasy Blood and Tissue (QBT), Macherey-Nagel’s NucleoMag DNA Microbiome (MN), Zymo Research’s ZymoBIOMICS DNA Mini Prep Kit (ZMI) and Qiagen’s DNeasy PowerSoil/QIAamp PowerFecal Pro (PS). The composition of Zymo Research’s ZymoBIOMICS MCS is shown on the far left (expected). Species are coloured according to the legend; Gram-negatives are shades of pink, Gram-positives are shades of blue and yeast species are shades of green.

Community composition was evaluated in two ways: (1) an MIQ score (Tables 1 and S4) and (2) the percent of total reads assigned to each species (Fig. 2, Table S4). MIQ scores closer to 100 indicate the methods that are best able to extract DNA from community members in proportion to their expected relative abundance [35, 37]. ZMI and PS most closely resembled the expected composition with mean MIQ scores of 81 and 67, respectively (Table 1, Fig. 2). The bacterial species in the MCS, each expected at 12%, were generally well represented with both methods, although *L. fermentum* and *P. aeruginosa* were slightly underrepresented (7.9–9.9%), and *E. coli* was overrepresented at ~17% (Fig. 2, Table S4). For the yeast species, expected at 2%, ZMI outperformed PS, particularly for *C. neoformans*, with 1.8% vs. 0.4% representation, respectively (Fig. 2, Table S4). QBT, NG and MN showed strong skewing towards Gram-negative species, accounting for ~80% of reads, despite representing only 36% of the MCS; this was reflected in MIQ scores of ≤5 for all replicates from these methods (Fig. 2, Table S4). ZMG showed less bias, with Gram-negatives comprising 60% of the reads, corresponding to an MIQ score of 33 (Table 1, Fig. 2).

Since long-read generation is a key advantage of ONT sequencing over other technologies [7–9], several read length parameters were generated (Table S4). However, only read length N50 values were examined in depth, as they provide more meaningful information than mean or median read length or longest read. Mean read length N50 values ranged from 4.5 to 16.5 kb (Table 1). ZMI produced the shortest values (4.5 kb), followed by PS (7.5 kb) and NG (9.8 kb, Table 1). MN and QBT yielded values in the 13–14 kb range, while ZMG allowed for the generation of the most long reads, with a mean of 16.5 kb (Table 1). Read length N50 values for replicates extracted with the same method were on the same order of magnitude (Table S4).

**Fig. 3.**
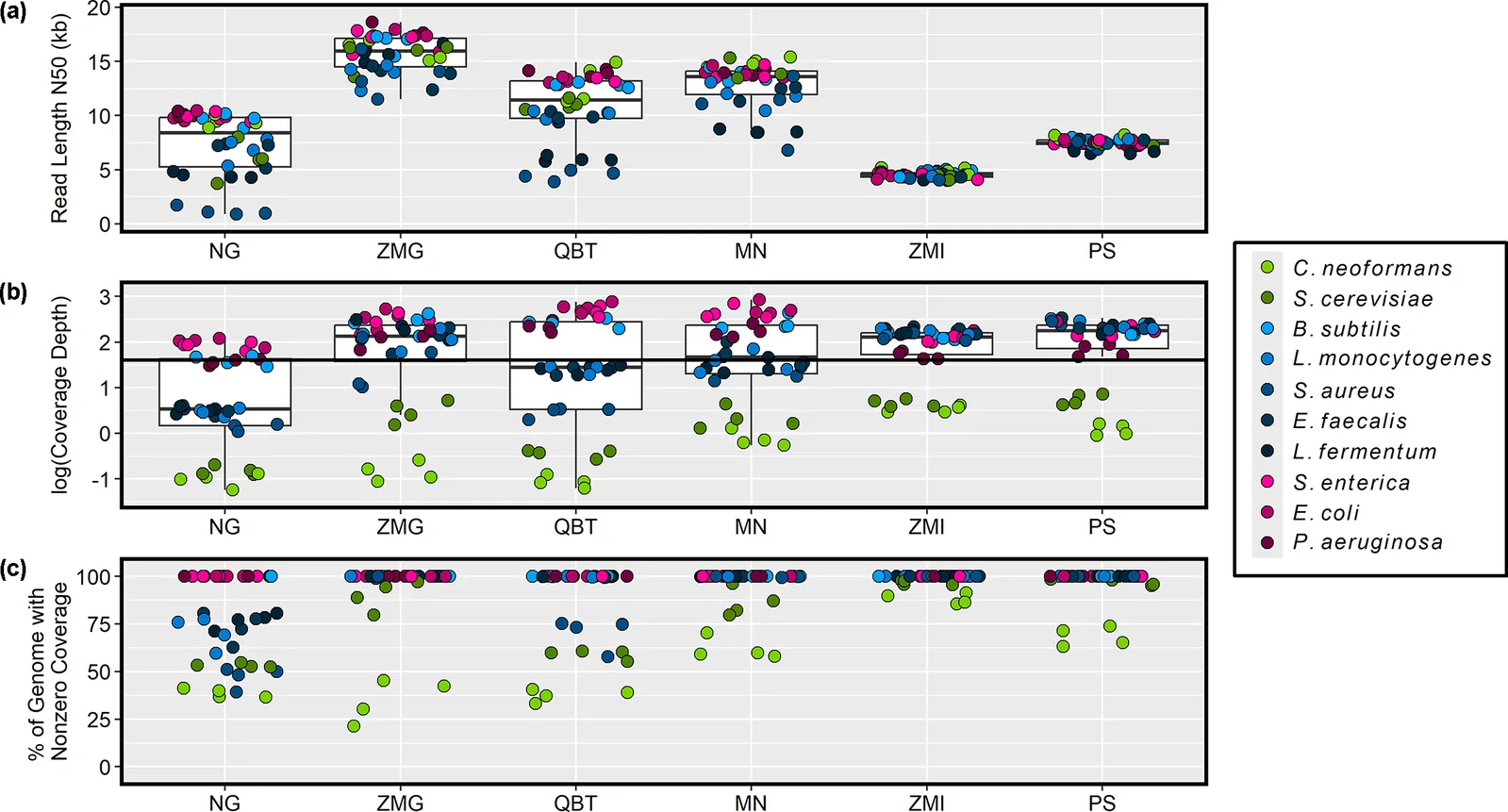
Read length N50 (**a**), log(coverage depth) (**b**) and the percentage of the genome with nonzero coverage (**c**) by species for each of the six extraction methods sequenced. The kits tested were Norgen BioTek Corp’s Stool DNA Isolation (NG), Zymo Research’s ZymoBIOMICS Quick-DNA HMW MagBead (ZMG), Qiagen’s DNeasy Blood and Tissue (QBT), Macherey-Nagel’s NucleoMag DNA Microbiome (MN), Zymo Research’s ZymoBIOMICS DNA Mini Prep Kit (ZMI) and Qiagen’s DNeasy PowerSoil/QIAamp PowerFecal Pro (PS). Species are coloured according to the legend; Gram-negatives are shades of pink, Gram-positives are shades of blue, and yeast species are shades of green. In panel (b), the black horizontal line indicates 40× coverage, and the y-axis is a log10 scale.

Read length N50 was also determined for each species within a replicate (Fig. 3a, Table S5). For each replicate, the difference between the species with the highest and lowest read length N50 was calculated, and the mean of these differences was then evaluated for each method (Tables 1 and S4). There was large variability (4.1 to 9.7 kb) between species values for ZMG, MN, NG and QBT, whereas ZMI and PS showed less variability (0.7 to 1.4 kb) (Table 1, Fig. 3a, Table S5). Methods with low interspecies variability (ZMI, PS) also showed less overall variability in read length N50 values (Fig. 3a, Table S5). In most cases, Gram-negative species had read length N50 values equal to or greater than the mean value for their respective method (Fig. 3a, Table S5).

### Genome metrics

Genome metrics were evaluated for reference-mapped reads and *de novo* assemblies, including depth and breadth of coverage, genome fraction, mismatches, LGA50, number of contigs per assembly and plasmid coverage. Where *k*-mer-based and alignment-based breadth of coverage metrics differed, *k*-mer-based breadth was treated as more relevant for method-level comparison and alignment-based breadth as more informative for localizing gaps.

The mean number of bacterial species (*n*=8) with greater than 40× coverage across the reference genome ranged from 3.0 to 8.0 (Table 1, Fig. 3b). Across all replicates of ZMI and PS, all eight bacterial species had greater than 40× coverage (Table 1, Fig. 3b, Tables S4 and S5). ZMG, MN, QBT and NG showed greater variability between replicates, resulting in means of 7.5, 5.3, 4.0 and 3.0 species with greater than 40× coverage, respectively. Most of these species were Gram-negative (Table 1, Fig. 3b, Table S5).

When using *k*-mer-based analysis to evaluate breadth of coverage, MN, ZMI and PS achieved at least 1× coverage across more than 99% of the genome for all eight bacterial genomes (Table 1, Fig. 3c, Tables S4 and S5). One replicate each from ZMI and PS had four species with 100% genome coverage (Tables S4 and S5). ZMG had three replicates achieving greater than 99% coverage for all eight bacterial genomes, and the fourth replicate fell just short for *S. aureus* (Fig. 3c, Table S5). In addition, all replicates from MN, ZMI, PS and ZMG had fewer than 60 gaps in any one genome when reads were assessed with an alignment-based approach (Fig. S1). In contrast, NG replicates did not achieve 1× coverage across 99% of the genomes for *L. fermentum*, *E. faecalis*, *S. aureus* or *L. monocytogenes*, and those chromosomes consistently had more than 1,000 gaps (Fig. 3c, Table S5, Fig. S1). QBT replicates similarly failed to reach 99% coverage breadth for *S. aureus*, which is also reflected in the fact that replicates had over 6,000 gaps in the *S. aureus* chromosome (Fig. 3c, Table S5, Fig. S1). Finally, for the yeast species, no method was able to achieve at least 1× coverage across 99% of either genome; however, two PS replicates reached greater than 98% 1× coverage for *S. cerevisiae* (Fig. 3c, Table S5).

There are seven plasmids present in the MCS: one each in *B. subtilis*, *E. coli* and *P. aeruginosa* genomes and two each in *S. enterica* and *S. aureus* genomes. When evaluating using a *k*-mer-based approach, all methods had 100% coverage for *B. subtilis*, *E. coli*, *P. aeruginosa* and *S. enterica* plasmids (Table S6). ZMG, MN, ZMI and PS methods also had 100% coverage of the plasmids attributed to *S. aureus*, while NG and QBT methods were unable to achieve this level of completeness (Table S6). For the larger of the two *S. aureus* plasmids (5,925 bp), NG achieved greater than 99% nonzero coverage in all replicates, whereas QBT only had one replicate reach this mark (Table S6). Both methods had less coverage for the smaller *S. aureus* plasmid (4748 bp), with QBT outperforming NG (Table S6). Coverage results from read mapping methods show some variation due to approach but also demonstrate that reads from ZMI and PS were consistently able to cover all regions of the *S. aureus* plasmids (Fig. S1).

*De novo* MAGs of the bacterial species were evaluated to determine genome fraction, LGA50, mismatches and total number of contigs. Mean genome fractions ranged from 72.5 to 97.8% (Table 1). ZMG, MN, ZMI and PS all had 97% or higher coverage of the eight bacterial species; however, only ZMG, MN and PS also had more than seven species with LGA50 of ≤1 contig (Tables 1 and S5). The mean number of mismatches between reference genome and assemblies ranged from 2.2 to 50.1 per 100 kb (Table 1). The mean number of total contigs for all bacterial species ranged from 15 to 93 across all methods, with NG and ZMI having the highest counts, 93 and 56, respectively (Table 1). In contrast, ZMG, MN and PS had mean mismatches of 3 or fewer per 100 kb and mean bacterial contigs counts below 25 (Table 1). Alignment of MAGs to the bacterial reference genomes showed comparable results, with ZMG, MN and PS yielding fewer gaps of greater than ten bases compared to the other methods (Fig. S2).

## Discussion

Previous ONT metagenomic studies reached conclusions about DNA extraction protocols based on single use cases with artificial communities or clinical samples [18–20]. In contrast, this study provides broader guidance by evaluating extraction methods encompassing a range of lysis and purification strategies and by using a matrix-free, commercially available standardized mock community with diverse cell wall architectures. This approach enables reproducibility and cross-study comparison [47]. By design, this study offers a framework within which results from testing other methods can be interpreted. Organizing the discussion by use case further assists readers in selecting the most appropriate methods for evaluation within their own laboratory. Among six DNA extraction methods, magnetic bead purification (MN, ZMG) produced the longest mean read length N50 values, while mechanical lysis was a key factor in preserving community composition accuracy for PS and ZMI.

### Use cases

#### Maximizing genome coverage and assembly completeness

The top performing methods for genome coverage and assembly completeness were ZMG and PS, measured by genome coverage across species, assembly completeness, plasmid recovery and proportional genome coverage across species (Table 1). Top performance is likely due to a combination of long reads, sequencing yield and representative community lysis. ZMG achieved the highest mean read length N50 values of all methods, whereas PS had one of the lowest (Table 1). This difference likely reflects methodology: enzyme-based lysis and magnetic beads (ZMG), versus mechanical lysis via bead-beating and a silica column (PS). Methods that used either enzymatic-lysis (QBT) or magnetic beads for DNA purification (MN) generated longer mean read length N50 values (Table 1), consistent with previous studies linking these methodologies to longer reads [13, 18–20]. Longer reads have been shown to improve assembly quality [13, 14], which likely explains ZMG’s strong performance; however, PS’s success suggests this is not the sole requirement for high-quality assemblies. Sequencing yield likely also contributes, as both ZMG and PS generated ample data and good quality scores (Table 1, Fig. 1b). When choosing between ZMG and PS, laboratories should consider additional metrics (Table 1) and the genomic features of their organism; ZMG’s longer reads would be advantageous for assembly of highly repetitive or recombinogenic genomes [13].

Some methods (ZMI, MN) performed well for either genome coverage or assembly, but not both. ZMI achieved good depth and breadth of coverage; however, its assemblies contained high numbers of mismatches and contigs, likely reflecting its shorter DNA fragments (Table 1), which is consistent with findings from Kruasuwan *et al*. [14]. MN showed strong assembly performance, likely due in part to its ability to generate long reads (Table 1), but was less successful at genome coverage across all species, probably because DNA was not extracted representatively from all community members (Figs 2 and 3b). This further supports the idea that a combination of read length, sequencing yield and representative community lysis impacts genome coverage and metagenomic assembly success.

#### Preserving composition accuracy

The most accurate community compositions were achieved with PS and ZMI, likely reflecting more effective lysis, as both methods utilize bead-beating. However, bead beating alone is insufficient to explain lysis efficiency. PS has a bead-beating duration similar to NG and MN, yet those methods had poorer community composition accuracy, while ZMG outperformed NG and MN despite lacking a bead-beating step (Table 1). Together, these observations demonstrate that although mechanical lysis can increase metagenomic diversity [16], it is not the sole determinant of lysis efficiency.

Lysis efficiency likely reflects a combination of bead size and material, lysis chemistry and enzymes and physical conditions such as temperature. ZMI’s composition accuracy may relate to its use of smaller beads; however, limited information about the size and material of beads for most methods makes comprehensive interpretation difficult. Moreover, ZMI and PS lack enzymatic lysis or static incubation steps that are present in all other methods, underscoring that increased lysis complexity does not guarantee improved composition accuracy.

As hypothesized, improved composition accuracy coincided with shorter reads; however, more vigorous lysis steps did not disproportionately shorten reads for easily lysed species as had been expected. ZMI and PS had the shortest mean read length N50 values with the lowest variability between species (Table 1, Fig. 3a), agreeing with the findings of Zhang *et al.* [19]. One possible explanation for the low read length N50 values of lysis-resistant species in other methods is that little or no lysis of these species occurred during extraction, and reads assigned to these species originated from cells that lysed during storage [48, 49].

#### Targeting specific species

The MCS contains species with a range of cell wall architectures, providing a useful baseline for evaluating DNA recovery. However, the absence of representatives from *Actinomycetota* and *Bacteroidota*, which are frequently found in natural communities, limits the ability to accurately predict extraction performance for species from these phyla.

NG, QBT and MN recovered approximately twice the expected proportion of reads from Gram-negative species (Fig. 2), making them suitable options when targets are exclusively Gram-negative and accurate community composition is not required. Among these methods, NG produced poorer assembly metrics than QBT and MN, which is expected considering its lower DNA and sequencing yields (Table 1). Given that MN is marketed for soil, stool and biofilm, it may be preferable to QBT for laboratories with more complex matrices in this use case.

To ensure sufficient extraction of Gram-positive or yeast species, ZMI or PS are the most appropriate (Fig. 2). Yeast recovery varied between the methods and the species, which is consistent with previous findings [17]. Both methods were able to generate high breadth and modest depth of genome coverage for *S. cerevisiae*. ZMI showed a similar pattern for *C. neoformans*, while PS was less consistent between yeast species (Fig. 3b, c).

#### Limiting required resources

Method choice may depend on equipment availability, time or budget constraints. All methods require micropipettes, a centrifuge with speed control and a timer. Bead-beating methods (NG, MN, ZMI and PS) require a vortex with a horizontal adaptor; magnetic bead purification methods (MN, ZMG) require a magnet; and some methods (NG, ZMG, QBT and MN) require a heat source. For fieldwork, ZMI and PS are quick and require minimal equipment. PS has previously shown strong performance in clinical settings [20], has short turnaround times and is available in an automatable 96-well plate format compatible with the QIAcube HT instrument [50]. NG and QBT are the least expensive options; however, their performance metrics limit suitability for certain use cases. Additionally, NG consistently generated low DNA and sequencing yields, which may be problematic if laboratories are interested in low-abundance species [6, 15].

### ONT recommendations

Of the three methods that did not yield sufficient DNA to meet current ONT recommendations of 200 ng in 10 µl [31], QIA was not sequenced due to insufficient yield under previous guidelines [32]. NG and MN achieved this cutoff and were sequenced with mixed results (Table 1, Fig. 1a). Although poor NG sequencing output supports ONT’s increased recommendation, MN’s performance suggests that it may be worth sequencing samples with as little as 100 ng of DNA available.

DNA absorbance ratio recommendations from ONT were not met by any replicates in this study (Fig. 1b). Consistently high 260/280 absorbance ratios had no apparent effect on sequencing output (Table 1), as has been previously reported [12]. Despite 260/230 absorbance ratios below the recommended minimum threshold (Fig. 1b), all methods achieved similar sequence read quality scores (Table 1), in agreement with previous reports [18, 51]. These results suggest that additional concentration or purification steps prior to sequencing may be unnecessary, even when concentrations and/or absorbance ratios fall outside ONT-recommended ranges, which is especially relevant when samples are precious, long reads are desired or time is limited.

### Conclusion

The metrics and recommendations developed here can guide laboratories when selecting DNA extraction methods most suitable for metagenomics within their specific use case. This includes analytical objectives such as maximizing genome coverage and assembly completeness, preserving composition accuracy, targeting specific species or limiting required resources. For genome coverage and assembly, both ZMG and PS performed well, but ZMG is more time-consuming and showed lower composition accuracy, while PS produced shorter reads. If composition accuracy is the most important factor, ZMI may be preferred, although it was only marginally better than PS with reduced coverage and assembly quality. QBT and MN are good choices for those only interested in Gram-negative bacteria. ZMI and PS are top contenders for settings with limited equipment. PS is also well suited to a diagnostic lab where HTP can assist with reducing hands-on and turnaround times. NG or QBT may be the top choice if the budget is restrictive, but laboratories need to be aware of possible limitations for downstream applications. Finally, although the MCS is a valuable tool for reproducibility testing, laboratories should confirm extraction efficiency for any method using their specific sample matrix and target organisms and review updates from the kit manufacturers and ONT, as offerings and guidelines can change.

## Supplementary material

10.1099/mgen.0.001738 Supplementary Material 1.

10.1099/mgen.0.001738 Supplementary Material 2.

## Abbreviations

CAD: Canadian dollars
Gbp: gigabases
HMW: high molecular weight
HTP: high-throughput
IHMS: International Human Microbiome Standards
KAT: K-mer Analysis Toolkit
MAG: metagenome-assembled genome
MCS: Microbial Community Standard
MIQ: Measurement Integrity Quotient
ONT: Oxford Nanopore Technologies
WGS: whole-genome sequencing
