# Supplementary material for: Benchmarking DNA extraction protocols across use cases for culture-independent Nanopore metagenomics
Source: Microb Genom. 2026 Jul 15;12(7):001738. doi: 10.1099/mgen.0.001738 (PMC13543651; doi:10.1099/mgen.0.001738)

**Fig. S1**: Heatmap of gaps from reads aligned to the reference genomes for each of the six extraction methods sequenced. The methods are ordered from top to bottom as follows: Norgen BioTek Corp's Stool DNA Isolation (NG); Zymo Research's ZymoBIOMICS Quick-DNA HMW MagBead (ZMG); Qiagen's DNeasy Blood and Tissue (QBT); Macherey-Nagel's NucleoMag DNA Microbiome (MN); Zymo Research's ZymoBIOMICS DNA Mini Prep Kit (ZMI); and Qiagen's DNeasy PowerSoil/QIAamp PowerFecal Pro (PS). For each method, the chromosome and associated plasmids are listed on the left. Gap-size categories (bp) are shown across the top, ranging from the shortest gaps on the left (1 bp) to the longest gaps (>1000 bp) on the right. Cell colour indicates the number of gaps detected, ranging from light blue (lowest) to red (highest), with raw counts overlaid within each cell. Blank cells indicate no gaps were detected. Replicates for each method are shown along the bottom.

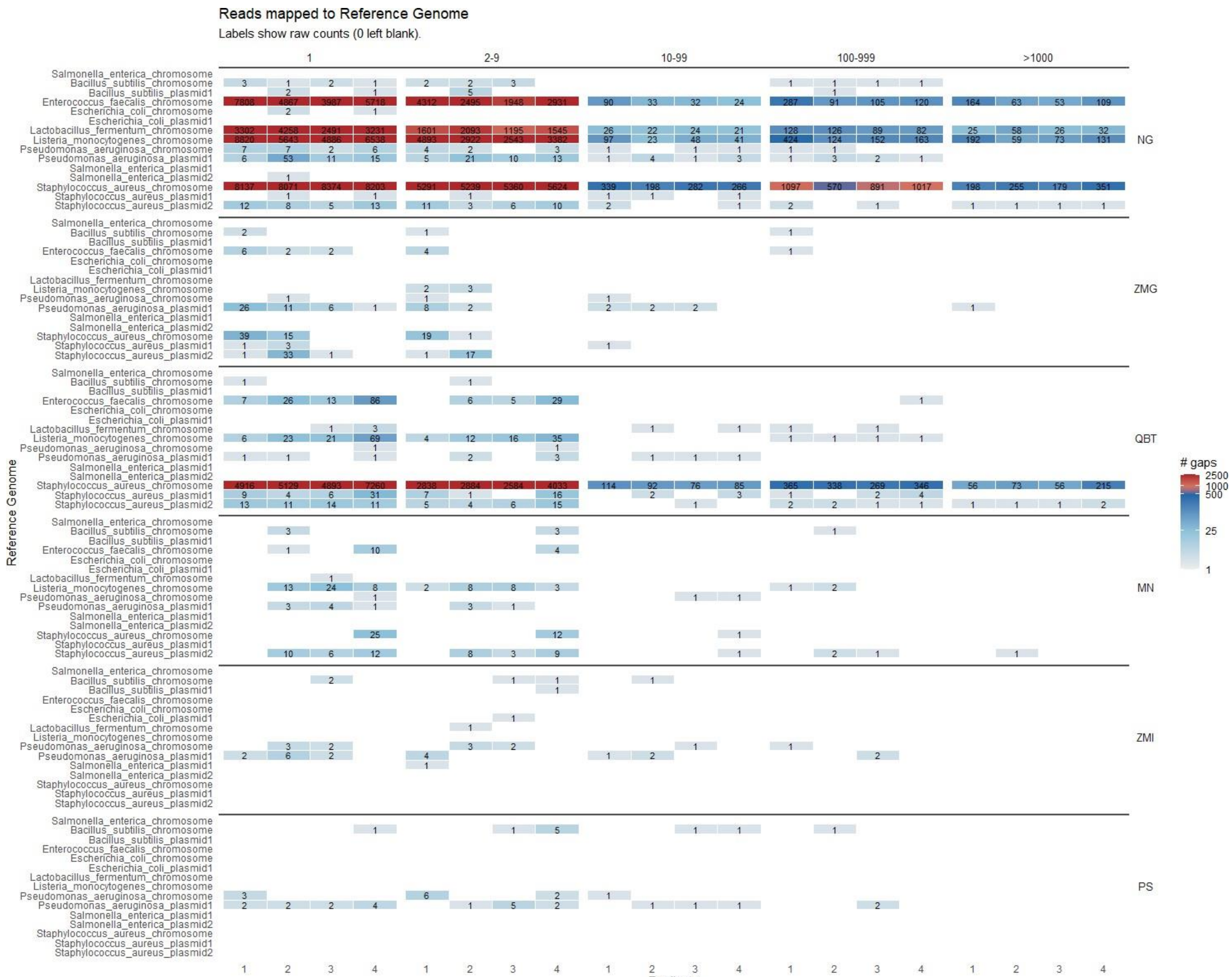

**Fig. S2.** Heatmap of gaps from assemblies aligned to the reference genomes for each of the six extraction methods sequenced. The methods are ordered from top to bottom as follows: Norgen BioTek Corp's Stool DNA Isolation (NG); Zymo Research's ZymoBIOMICS Quick-DNA HMW MagBead (ZMG); Qiagen's DNeasy Blood and Tissue (QBT); Macherey-Nagel's NucleoMag DNA Microbiome (MN); Zymo Research's ZymoBIOMICS DNA Mini Prep Kit (ZMI); and Qiagen's DNeasy PowerSoil/QIAamp PowerFecal Pro (PS). For each method, the chromosome and associated plasmids are listed on the left. Gap-size categories (bp) are shown across the top, ranging from the shortest gaps on the left (1 bp) to the longest gaps (>10000 bp) on the right. Cell colour indicates the number of gaps detected, ranging from light blue (lowest) to red (highest), with raw counts overlaid within each cell. Blank cells indicate no gaps were detected. Replicates for each method are shown along the bottom.

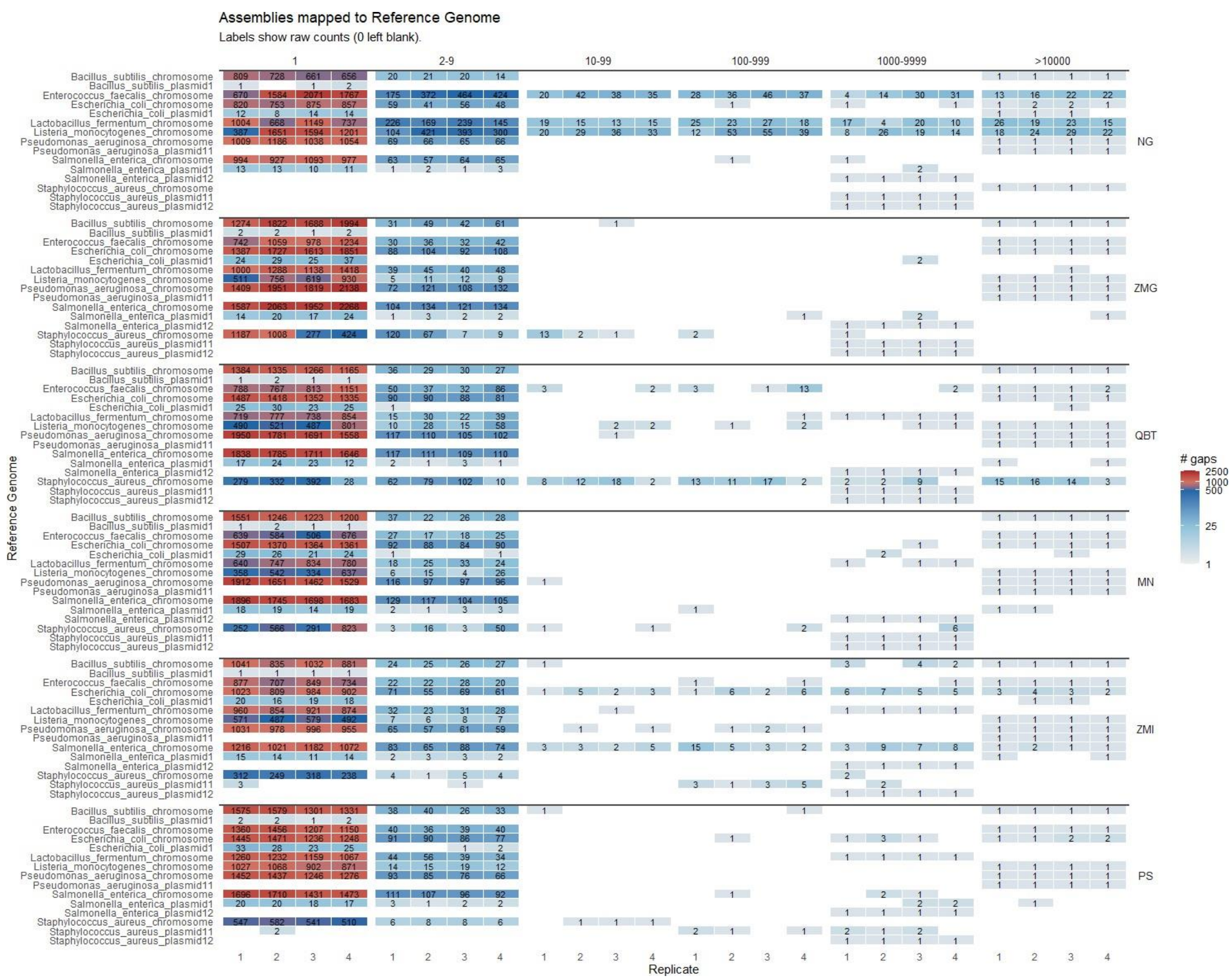

Supplement: Supplementary file 1 [file mgen-12-01738-s001.pdf]
